# Diabetes mellitus modifies the association between chronic kidney disease–mineral and bone disorder biomarkers and aortic stiffness in peritoneal dialysis patients

**DOI:** 10.1038/s41598-024-55364-3

**Published:** 2024-02-24

**Authors:** Hsiang-Jung Huang, Bang-Gee Hsu, Chih-Hsien Wang, Jen-Pi Tsai, Yi-Hsin Chen, Szu-Chun Hung, Yu-Li Lin

**Affiliations:** 1 Department of Internal Medicine, Hualien Tzu Chi Hospital, Buddhist Tzu Chi Medical Foundation, Hualien, 97004 Taiwan; 2 School of Medicine, Tzu Chi University, Hualien, 97004 Taiwan; 3 Division of Nephrology, Hualien Tzu Chi Hospital, Buddhist Tzu Chi Medical Foundation, Hualien, 97004 Taiwan; 4 Division of Nephrology, Department of Internal Medicine, Dalin Tzu Chi Hospital, Buddhist Tzu Chi Medical Foundation, Chiayi, 62247 Taiwan; 5 Division of Nephrology, Department of Internal Medicine, Taichung Tzu Chi Hospital, Buddhist Tzu Chi Medical Foundation, Taichung, 40201 Taiwan; 6 Division of Nephrology, Department of Internal Medicine, Taipei Tzu Chi Hospital, Buddhist Tzu Chi Medical Foundation, Taipei, 23142 Taiwan

**Keywords:** Peritoneal dialysis, Diagnostic markers, Vascular diseases

## Abstract

This study aimed to investigate the relationship of four chronic kidney disease–mineral and bone disorder (CKD–MBD) biomarkers, including intact parathyroid hormone (PTH), fibroblast growth factor 23 (FGF23), soluble klotho, and fetuin-A, with aortic stiffness in peritoneal dialysis (PD) patients, comparing those with and without diabetes mellitus (DM). A total of 213 patients (mean age 58 ± 14 years; 81 (38.0%) patients with DM) were enrolled. Their aortic pulse wave velocity (PWV) was measured using pressure applanation tonometry, while serum intact PTH, FGF23, α-klotho, and fetuin-A levels were measured using enzyme-linked immunosorbent assay. Overall, patients with DM had higher aortic PWV than those without (9.9 ± 1.8 vs. 8.6 ± 1.4 m/s, *p* < 0.001). Among the four CKD–MBD biomarkers, FGF23 levels were significantly lower in DM group (462 [127–1790] vs. 1237 [251–3120] pg/mL, *p* = 0.028) and log-FGF23 independently predicted aortic PWV in DM group (β: 0.61, 95% confidence interval: 0.06–1.16, *p* = 0.029 in DM group; β: 0.10, 95% confidence interval: − 0.24–0.45, *p* = 0.546 in nonDM group; interaction *p* = 0.016). In conclusion, the association between FGF23 and aortic PWV was significantly modified by DM status in PD patients.

## Introduction

The high prevalence of end-stage renal disease (ESRD) and its related cardiovascular burden are major public health concerns worldwide. Cardiovascular disease (CVD) is the leading cause of death in ESRD, accounting for half of all mortality in patients undergoing dialysis^1–3^. Aside from the well-established traditional risk factors, chronic kidney disease–mineral and bone disorder (CKD–MBD), a dysregulation of mineral and bone metabolism exacerbated by worsening kidney disease, plays a crucial role in the pathogenesis of CVD^4–6^.

Compensatory changes in several serum CKD–MBD biomarkers are observed as CKD progresses^7^. Fibroblast growth factor 23 (FGF23), secreted primarily by osteocytes and osteoblasts, is the earliest elevated biomarkers regulating mineral bone metabolism. Its serum levels increase 1000-fold when patients develop ESRD^8,9^. Through binding to klotho, FGF23 inhibits renal phosphorus reabsorption by downregulating sodium/phosphate cotransporters in the proximal renal tubules. It also regulates the secretion of parathyroid hormone (PTH), which levels are elevated in advanced CKD stages^10–13^. In contrast, circulating soluble α-klotho, the extracellular domain of membranous klotho proteolytically cleaved by metalloproteinase, is reduced in early CKD stages^7^. Fetuin-A, a 59-kDa glycoprotein derived from the liver, is a potent inhibitor of vascular calcification. Its serum levels have steadily decreased since the early stages of CKD^14,15^. The aforementioned biomarker changes may be involved in the pathogenesis of distinctive vascular dysfunction in CKD.

As the most remarkable alteration in these serum CKD–MBD markers is observed at the ESRD stage, assessing their association with arterial stiffness in patients undergoing peritoneal dialysis (PD) is essential. Although dysregulated mineral metabolism is more severe in patients with diabetes mellitus (DM) than in those without^16,17^, it is unknown whether DM status modifies the association between serum CKD–MBD biomarkers and aortic stiffness.

Thus, we aimed to investigate the relationship of serum CKD–MBD biomarkers, including intact PTH, FGF23, soluble α-klotho, and fetuin-A, with aortic stiffness in patients with ESRD undergoing PD, comparing those with and without DM.

## Materials and methods

### Patients

In this cross-sectional study, the association of serum CKD–MBD biomarkers with aortic stiffness in PD patients with and without DM was investigated. Patients with ESRD who had undergone PD for more than three months were screened at Hualien Tzu Chi Hospital, a tertiary medical center in eastern Taiwan, and its three branch hospitals in Taipei, Taichung, and Dalin. Patients were enrolled and baseline measurements were taken between February 2020 and May 2021. Patients who had an infection, acute myocardial infarction, decompensated heart failure, stroke, or amputation at the time of enrollment, or who refused to participate, were excluded from the study. The study was approved by Tzu Chi University and Hospital’s Institutional Review Board for the Protection of Human Subjects (IRB no. 108-219-A), and all participants provided informed consent before participating in this study. All methods were performed in accordance with the relevant guidelines and regulations.

The basic information, including age, gender, PD vintage, dialysis modalities, comorbidities, and medications used, was obtained from the electronic medical records. The diagnosis of DM was identified using the ICD-10 code from the electronic medical records.

### Anthropometric and aortic pulse wave velocity measurements

A well-trained staff measured participants’ height and weight while they were barefoot and wearing light clothing, and their body mass index was calculated accordingly.

The aortic pulse wave velocity (PWV) was measured using a cuff-based volumetric displacement (SphygmoCor XCEL, AtCor Medical, Sydney, NSW, Australia)^18^. Briefly, the cuff of the XCEL device was placed on the participants’ left upper arm to measure blood pressure (BP) with an automatic recording of standard oscillometric brachial systolic and diastolic BP, immediately followed by reinflation of the cuff to a sub diastolic pressure level. For PWV assessment, the XCEL device uses the volumetric displacement waveform from a cuff around the upper thigh instead of femoral artery tonometry, and a tonometry is used in the XCEL device for carotid pulse acquisition. Pulse pressure was calculated by subtracting the brachial diastolic BP from the brachial systolic BP.

### Biochemical investigations

Fasting blood samples were collected in the morning and centrifuged at 3000 × *g* for 10 min to separate serum samples, which were biochemically analyzed within one hour of collection. Serum levels of blood urea nitrogen, creatinine, glucose, albumin, total calcium, phosphorus, and total alkaline phosphatase were measured using an autoanalyzer (Siemens Advia 1800, Siemens Healthcare GmbH, Henkestr, Germany). Corrected calcium levels, calculated as total calcium (mg/dL) + 0.8 [4—serum albumin (mg/dL)], were adopted for analysis. Furthermore, 24-h urine and dialysate samples were collected to calculate weekly peritoneal and renal creatinine clearance^19^. Those who had residual urine output were defined as having preserved residual renal function.

Serum CKD–MBD biomarkers were quantified using commercial enzyme-linked immunosorbent assays for intact PTH (IBL International GmbH, Hamburg, Germany), FGF23 (C-terminal, Immutopics, Inc., San Clemente, CA), soluble α-klotho (Immuno-Biological Laboratories Co., Ltd., Fujioka-Shi, Gunma, Japan), and fetuin-A (BioVender Laboratory Medicine Inc., Modrice, Czech Republic).

### Statistical analysis

Continuous data were assessed for normality using the Kolmogorov–Smirnov test. Normally distributed data were expressed as means and standard deviations and compared using the Student’s independent *t*-test, while nonnormally distributed data were expressed as medians and interquartile ranges and compared using the Mann–Whitney *U* test between DM and nonDM groups. Categorical data were expressed as numbers and percentages and compared using the χ^2^ test. The correlation of serum CKD–MBD biomarkers levels with clinical parameters and aortic PWV were analyzed using Pearson’s or Spearman’s coefficient analysis, as appropriate. Serum CKD–MBD biomarkers showed a right-skewed distribution and were log-transformed before regression analysis. A multivariate stepwise linear regression analysis was performed to determine the independency between CKD–MBD biomarkers and aortic PWV stratified by DM status. The potential modified effects of DM status on the association between CKD–MBD biomarkers and aortic PWV effects were tested by creating DM × mean-centering biomarkers interaction variables. Data were analyzed using the Statistical Package for the Social Sciences (SPSS) for Windows (Version 19.0; SPSS Inc., Chicago, IL, USA). *P*-values of less than 0.05 were considered statistically significant.

## Results

This study enrolled 213 patients undergoing PD, 38.0% of whom (*n* = 81) had DM and 62.0% (*n* = 132) did not. Table 1 summarizes the clinical characteristics of patients undergoing PD. The mean age was 58 ± 14 years, and the median PD vintage was 48 months. Among these patients, 55.4% were female, 91.1% had hypertension, 46.0% had hyperlipidemia, 33.8% received continuous ambulatory PD, and 57.3% underwent automated PD. Calcium carbonate, active vitamin D, and statins were used by 69.0%, 22.1%, and 31.0% of patients, respectively.

**Table 1:** Characteristics of the study population.

Characteristic	All participants (*n* = 213)	DM group (*n* = 81)	NonDM group (*n* = 132)	*P*-value
Basic information				
Age (years)	58 ± 14	60 ± 11	57 ± 15	0.051
Female, *n* (%)	118 (55.4)	39 (48.1)	79 (59.8)	0.095
PD vintage (months)	48 (21–82)	33 (17–62)	55 (27–92)	0.002*
Dialysis modalities				
CAPD, *n* (%)	72 (33.8)	30 (37.0)	42 (31.8)	0.106
APD, *n* (%)	122 (57.3)	48 (59.3)	74 (56.1)	
Residual renal function, *n* (%)	122 (57.3)	53 (65.4)	69 (52.3)	0.060
Hypertension, *n* (%)	194 (91.1)	76 (93.8)	118 (89.4)	0.270
Hyperlipidemia, *n* (%)	98 (46.0)	41 (50.6)	57 (43.2)	0.291
Calcium carbonate, *n* (%)	147 (69.0)	56 (69.1)	91 (68.9)	0.976
Active vitamin D, *n* (%)	47 (22.1)	13 (16.0)	34 (25.8)	0.097
Statins, *n* (%)	66 (31.0)	30 (37.0)	36 (27.3)	0.135
Clinical data				
BMI (kg/m^2^)	25.0 ± 4.1	26.3 ± 4.0	24.1 ± 4.0	< 0.001*
Systolic BP (mmHg)	149.9 ± 22.4	157.3 ± 20.0	145.5 ± 22.8	< 0.001*
Diastolic BP (mmHg)	85.0 ± 14.4	84.8 ± 12.2	85.1 ± 15.7	0.877
Pulse pressure (mmHg)	47.5 ± 21.5	53.8 ± 17.9	43.7 ± 22.7	0.001*
Aortic PWV (m/s)	9.1 ± 1.7	9.9 ± 1.8	8.6 ± 1.4	< 0.001*
Laboratory data				
Peritoneal CCr (L/week)	47 (38–55)	45 (40–55)	48 (37–55)	0.899
Renal CCr (L/week)	3 (0–18)	7 (0–27)	1 (0–18)	0.035*
Glucose (mg/dL)	102 (92–124)	135 (108–168)	96 (89–105)	< 0.001*
Albumin (g/dL)	3.6 (3.3–3.8)	3.6 (3.3–3.8)	3.6 (3.4–3.8)	0.696
Calcium (mg/dL)	9.6 ± 0.7	9.7 ± 0.7	9.6 ± 0.8	0.333
Phosphorus (mg/dL)	5.2 ± 1.4	5.0 ± 1.2	5.3 ± 1.4	0.085
Calcium × phosphorus (mg^2^/dL^2^)	49.9 ± 13.5	48.2 ± 11.8	51.0 ± 14.5	0.131
Total ALP (U/L)	84 (61–112)	87 (60–109)	82 (61–114)	0.658
CKD–MBD biomarkers				
Intact PTH (pg/mL)	239 (96–479)	206 (78–341)	259 (114–546)	0.124
FGF23 (pg/mL)	708 (199–2771)	462 (127–1790)	1237 (251–3120)	0.028*
α-Klotho (pg/mL)	624 (384–903)	661 (443–922)	608 (373–890)	0.256
Fetuin-A (µg/mL)	323 (254–405)	312 (240–385)	328 (257–426)	0.236

Continuous variable values are presented as mean ± standard deviation and were analyzed using Student’s *t*-test; nonnormally distributed variable values are presented as medians and interquartile ranges and were analyzed using the Mann–Whitney *U* test; and categorical variables are presented as numbers (percentages) and were analyzed using the chi-squared test.

*DM* diabetes mellitus, *PD* peritoneal dialysis, *CAPD* continuous ambulatory peritoneal dialysis, *APD* automated peritoneal dialysis, *BMI* body mass index, *BP* blood pressure, *PWV* pulse wave velocity, *CCr* creatinine clearance, *calcium* × *phosphorus* calcium-phosphorus product, *ALP* alkaline phosphatase, *PTH* parathyroid hormone, *FGF23* fibroblast growth factor 23.

*A *p*-value of less than 0.05 was considered statistically significant.

Compared to patients without DM, patients with DM were older, had a shorter PD vintage, more preserved residual renal function, and higher body mass index, systolic BP, pulse pressure, and aortic PWV (9.9 ± 1.8 vs. 8.6 ± 1.4 m/s, *p* < 0.001). Notably, among four CKD–MBD biomarkers, patients with DM had significantly lower FGF23 levels (462 [127–1790] vs. 1237 [251–3120] pg/mL, *p* = 0.028). However, the serum levels of the other three biomarkers were comparable between groups.

Table 2 presents the simple correlations of log-transformed CKD–MBD biomarkers levels with clinical and laboratory parameters in patients with and without DM. In patients with DM, PD vintage was positively correlated with intact PTH (*r* = 0.31) and FGF23 (*r* = 0.33), while renal creatinine clearance was negatively correlated with FGF23 (*r* = − 0.28). In both patients with and without DM, serum FGF23 was positively correlated with calcium (*r* = 0.36 and 0.31), phosphorus (*r* = 0.27 and 0.45), and calcium × phosphorus (*r* = 0.37 and 0.52), while fetuin-A was positively correlated with albumin (*r* = 0.27 and 0.25). For biomarker intercorrelation, FGF23 was positively correlated with intact PTH (*r* = 0.23) and α-klotho (*r* = 0.25), while α-klotho was positively correlated with fetuin-A (*r* = 0.38) in patients with DM. However, similar intercorrelations among CKD biomarkers were not observed in patients without DM.

**Table 2:** Simple correlation analysis of relevant clinical factors with serum chronic kidney disease–mineral and bone disorder biomarkers in peritoneal dialysis patients with or without diabetes mellitus.

Variables	Correlation* coefficient*			
	Log-intact PTH (pg/mL)	Log-FGF23 (pg/mL)	Log-α-klotho (pg/mL)	Log-fetuin-A (µg/mL)
DM group (*n* = 81)				
Age (years)	0.03	− 0.08	0.02	− 0.01
PD vintage (months)^a^	0.31**	0.33**	− 0.02	− 0.01
Clinical data				
BMI (kg/m^2^)	− 0.08	0.08	0.01	− 0.07
Systolic BP (mmHg)	− 0.09	0.13	0.11	0.01
Diastolic BP (mmHg)	− 0.19	0.01	0.16	0.08
Pulse pressure (mmHg)	0.01	0.07	− 0.01	− 0.09
Peritoneal CCr (L/week)^a^	0.09	0.29**	0.06	− 0.08
Renal CCr (L/week)^a^	− 0.17	− 0.28*	− 0.18	0.11
Laboratory data				
Glucose (mg/dL)^a^	− 0.04	0.16	0.05	− 0.14
Albumin (g/dL)^a^	− 0.13	0.09	0.17	0.27*
Calcium (mg/dL)	− 0.11	0.36**	0.05	0.11
Phosphorus (mg/dL)	0.06	0.27*	0.06	0.13
Calcium × phosphorus (mg^2^/dL^2^)	0.03	0.37**	0.08	0.16
Total ALP (U/L)^a^	0.29**	0.16	0.07	− 0.23*
CKD–MBD biomarkers				
Log-intact PTH (pg/mL)	–	0.23*	− 0.02	0.01
Log-FGF23 (pg/mL)	0.23*	–	0.25*	0.15
Log-α-klotho (pg/mL)	− 0.02	0.25*	–	0.38**
Log-fetuin-A (µg/mL)	0.01	0.15	0.38**	–
NonDM group (*n* = 132)				
Age (years)	0.01	− 0.23**	0.01	− 0.06
PD vintage (months)^a^	0.01	0.15	− 0.08	− 0.07
Clinical data				
BMI (kg/m^2^)	0.11	0.22*	− 0.07	0.04
Systolic BP (mmHg)	0.04	0.05	0.09	− 0.10
Diastolic BP (mmHg)	− 0.02	0.05	0.09	− 0.05
Pulse pressure (mmHg)	0.11	0.11	0.05	0.01
Peritoneal CCr (L/week)^a^	0.01	0.13	0.10	0.04
Renal CCr (L/week)^a^	0.05	− 0.14	0.03	0.10
Laboratory data				
Glucose (mg/dL)^a^	− 0.01	0.01	0.12	0.11
Albumin (g/dL)^a^	− 0.04	0.09	− 0.07	0.25**
Calcium (mg/dL)	0.26**	0.31***	0.15	0.04
Phosphorus (mg/dL)	0.15	0.45***	− 0.07	− 0.15
Calcium × phosphorus (mg^2^/dL^2^)	0.24**	0.52***	− 0.02	− 0.14
Total ALP (U/L)^a^	0.08	− 0.14	0.05	− 0.17
CKD–MBD biomarkers				
Log-Intact PTH (pg/mL)	–	0.16	− 0.01	− 0.15
Log-FGF23 (pg/mL)	0.16	–	0.09	− 0.03
Log-α-klotho (pg/mL)	− 0.01	0.09	–	0.12
Log-fetuin-A (µg/mL)	− 0.15	− 0.03	0.12	–

^a^The variables had a nonnormal distribution and were analyzed using Spearman’s correlation. Otherwise, Pearson’s correlation was adopted.

*CKD–MBD* chronic kidney disease–mineral and bone disorder, *DM* diabetes mellitus, *PD* peritoneal dialysis, *BMI* body mass index, *BP* blood pressure, *CCr* creatinine clearance, *Calcium* × *phosphorus* calcium-phosphorus product, *ALP* alkaline phosphatase, *PTH* parathyroid hormone, *FGF23* fibroblast growth factor 23.

*P < 0.05, **p < 0.01, and ***p < 0.001.

**Figure 1:**
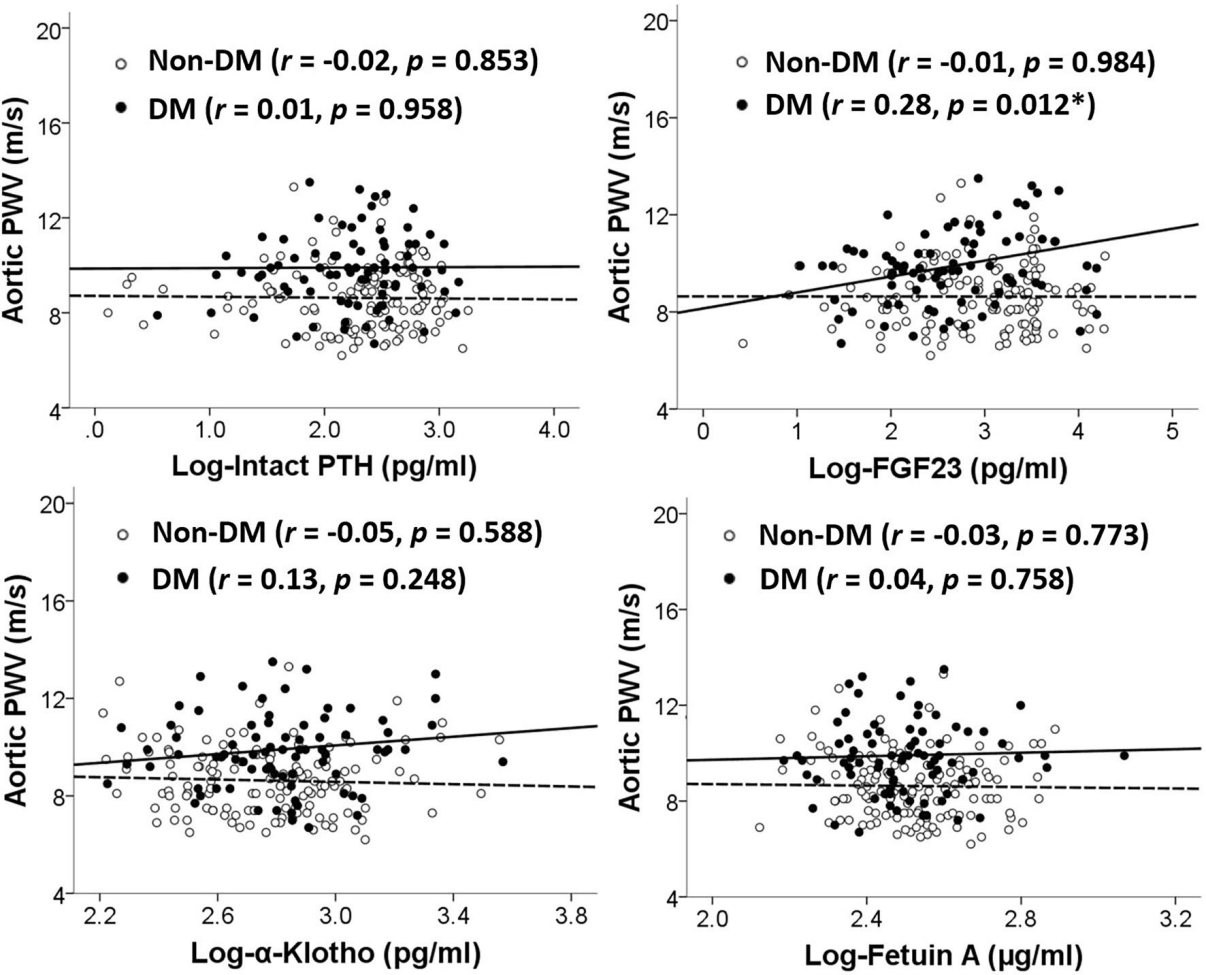
Scatter plots and Pearson’s correlation analysis of the association between serum chronic kidney disease–mineral and bone disorder biomarker levels and aortic pulse wave velocity values in peritoneal dialysis patients with and without DM.

Figure 1 depicts scatter plots and Pearson’s correlation analysis of the association between serum CKD–MBD biomarkers and aortic PWV in patients with and without DM. FGF23 had a positive correlation with aortic PWV in patients with DM (*r* = 0.28, *p* = 0.012) but not in those without (*r* = − 0.01, *p* = 0.984). The other three biomarkers were not associated with aortic PWV in patients with or without DM.

Table 3 shows the association of CKD–MBD biomarkers with aortic PWV in PD patients with and without DM using univariable and multivariable linear regression analysis. After adjusting for age, gender, PD vintage, residual renal function, hypertension, hyperlipidemia, alkaline phosphatase, calcium-phosphorus product, and calcium carbonate and active vitamin D use, log-FGF23 (β: 0.61, 95% confidence interval: 0.06–1.16, *p* = 0.029) was an independent predictor of aortic PWV in patients with DM but not in those without (β: 0.10, 95% confidence interval: − 0.24–0.45, *p* = 0.546). The DM status significantly modified the association between FGF23 and aortic PWV (interaction *p* = 0.016).

**Table 3:** A multivariate linear regression analysis of the association between chronic kidney disease–mineral and bone disorder biomarkers and aortic pulse wave velocity stratified by diabetes mellitus status.

Models	Aortic PWV (m/s)				*P*-value for interaction
	DM group		NonDM group		
	*β* (95% CI)	*p*	*β* (95% CI)	*p*	
Log-intact PTH (pg/mL)					
Unadjusted	0.02 (− 0.73–0.77)	0.958	− 0.04 (− 0.43–0.35)	0.853	0.885
Adjusted	− 0.50 (− 1.25–0.25)	0.187	− 0.09 (− 0.48–0.30)	0.652	0.845
Log-FGF23 (pg/mL)					
Unadjusted	0.66 (0.15–1.17)	0.012*	− 0.01 (− 0.31–0.31)	0.984	0.021*
Adjusted	0.61 (0.06–1.16)	0.029*	0.10 (− 0.24–0.45)	0.546	0.016*
Log-α-klotho (pg/mL)					
Unadjusted	0.89 (− 0.64–2.42)	0.248	− 0.24 (− 1.11–0.63)	0.588	0.172
Adjusted	0.69 (− 0.75–2.13)	0.341	− 0.15 (− 0.96–0.66)	0.712	0.363
Log-fetuin-A (µg/mL)					
Unadjusted	0.37 (− 2.00–2.74)	0.758	− 0.15 (− 1.18–0.88)	0.773	0.660
Adjusted	0.02 (− 2.35–2.40)	0.984	0.09 (− 0.91–1.08)	0.865	0.734

In the adjusted models, age, sex, PD vintage, residual renal function, hypertension, hyperlipidemia, alkaline phosphatase, calcium-phosphorus product, and calcium carbonate and active vitamin D use were all adopted as covariates.

*CI* confidence interval, *DM* diabetes mellitus, *PTH* parathyroid hormone, *FGF23* fibroblast growth factor 23.

## Discussion

This study investigated the association of four serum CKD–MBD biomarkers with aortic stiffness between PD patients with and without DM. The study’s novel finding is that serum FGF23 independently predicted aortic PWV only in PD patients with DM, albeit with lower serum FGF23 levels.

The close relationship between FGF23 and aortic stiffness in CKD and ESRD is explained by several mechanisms. FGF23 accelerates phosphate-induced calcification of vascular smooth muscle cells by stimulating osteoblastic differentiation^20^, activates the renin–angiotensin–aldosterone system^21^, and directly impairs endothelial vasorelaxation^22^. Notably, a recent study conducted by Vergara et al. showed that FGF23 treatment transformed vascular smooth muscle cells from a contractile to a synthetic phenotype in vitro, through the downregulation of microRNA-221/222 and phosphorylation of FGFR1 and Erk1/2^23^. Furthermore, FGF23 expression and serum levels are elevated in inflammatory states^24,25^. Several clinical observational studies have demonstrated associations between serum FGF23 levels and vascular stiffness at various stages of CKD. The large-scale Prospective Investigation of the Vasculature in Uppsala Seniors (PIVUS) study found that serum FGF23 was associated with arterial stiffness in community subjects with impaired renal function (estimated glomerular filtration rate less than 60 mL/min/1.73 m^2^)^26^. The association between serum FGF23 and total body atherosclerosis was also confirmed in a PIVUS subsample^27^. In patients on hemodialysis, serum FGF23 was associated with aortic calcification^28^ and accelerated the progression of coronary arterial calcification^29,30^. In patients undergoing PD, elevated serum FGF23 levels have been linked to vascular calcification^31^ and carotid artery intima-media thickness^32^. However, studies comparing the potentially different impacts of FGF23 on aortic stiffness in patients with and without DM are scarce.

In our study, serum FGF23 levels were positively associated with aortic stiffness in PD patients with DM but not in those without. Consistent with our finding, a significant positive association between serum FGF23 levels and aortic stiffness was found in non-CKD patients with type 1 DM but not in those with no DM^33^. In patients with coronary artery disease, serum FGF-23 levels predicted adverse cardiovascular outcomes only in those with type 2 DM but not in those without^34^. This suggested that DM status may modify FGF23’s effects on vascular pathology and clinical cardiovascular events. Although the underlying mechanisms remain unclear, some DM-specific vascular pathological factors, such as advanced glycosylated end products, insulin resistance, and reactive oxygen species, may interplay in the complex pathogenesis of CKD–MBD and act synergistically with FGF23 on changing vascular wall structure.

In the Predictors of Arrhythmic and Cardiovascular Risk in End Stage Renal Disease (PACE) cohort, primarily comprising incident hemodialysis patients of African American descent, elevated serum FGF-23 levels were associated with a higher prevalence of coronary artery calcification. However, contrary to our findings, higher FGF-23 levels were linked to lower baseline PWV and reduced PWV progression during follow-up, particularly among patients with DM^35^. The exact cause of these discrepancies remains unclear, but they might be attributed to differences in ethnic populations, patient selection, and study design.

Several DM-related factors, such as advanced glycated end product and glycerol-3-phosphate accumulation and enhanced chronic inflammation, are known to stimulate FGF23 secretion^16^. In patients with CKD who did not undergo dialysis, those with DM have higher FGF23 levels^17^ and more rapidly rising serum levels^36^ than those without DM. Surprisingly, our PD patients with DM had significantly lower serum FGF23 levels. A similar finding had been reported in the Japan Dialysis Outcomes and Practice Patterns study (J-DOPPS) and the Hemodialysis (HEMO) cohort, two large prevalent hemodialysis cohorts in Japan and the United States, respectively^37,38^. This finding could be attributed in part to patient selection. In these ESRD populations, patients with DM tended to have a shorter dialysis vintage and more preserved residual renal function, two major determinants of serum FGF23 levels^39,40^. Furthermore, a recent study demonstrated that insulin suppressed FGF23 production by inhibiting the transcription factor forkhead box protein O1^41^. Nevertheless, the positive correlation between relatively lower serum FGF23 levels and aortic PWV values in DM suggests that FGF23 is a more sensitive biomarker for aortic stiffness in PD patients with DM.

In our study, serum fetuin-A, a potent circulatory inhibitor of vascular calcification, was not associated with aortic stiffness in either the DM or nonDM groups. A similar finding was reported in two other dialysis cohorts^42,43^. As we all know, vascular calcification is a hallmark of CKD, but it is not the only factor contributing to aortic stiffness. Nevertheless, serum fetuin-A levels were found to be positively correlated with serum albumin levels in both PD patients with and without DM. High circulating fetuin-A levels reduced the inflammatory process, resulting in higher albumin levels^44,45^. Wang and colleagues found that lower serum fetuin-A levels in patients undergoing PD were associated with malnutrition, as assessed by serum albumin assay and subjective global assessment^46^.

Klotho is an antiaging protein, and its declining levels may be related to accelerated vascular aging. In the National Health and Nutrition Examination Survey (NHANES), a large cohort of the general population in the United States, serum klotho was found to be negatively associated with pulse pressure^47^. In contrast, in a general Chinese population, serum klotho levels did not predict BP or aortic PWV^48^. Similarly, in the KoreaN Cohort Study for Outcome in Patients With Chronic Kidney Disease (KNOW-CKD) cohort, serum klotho levels were not associated with brachial-to-ankle PWV in patients with advanced non-dialysis CKD^49^. In our patients undergoing PD, serum klotho was not associated with aortic PWV or pulse pressure. The discrepancy among these studies may be explained by differences in study populations and ethics.

The present study has several limitations that should be acknowledged. First, there was no image evaluation for aortic or coronary calcification, as well as other indices of vascular dysfunction, in this study. Second, neither vitamin D status nor inflammatory markers were assessed in this study. Third, the causal relationship between serum FGF23 and aortic stiffness cannot be established in this cross-sectional study, and longitudinal analyses are warranted to determine whether higher serum FGF23 levels contribute to accelerated aortic stiffness in patients with DM.

In conclusion, among CKD–MBD biomarkers, serum FGF23 was an independent predictor of aortic stiffness in PD patients with DM but not in those without DM. Further studies are needed to clarify the potential mechanisms underlying the effect of FGF23 and DM status interaction on aortic stiffness in patients undergoing PD.

## Data Availability

The datasets analysed during the current study are available from the corresponding author on reasonable request.
